# Genetic Causes of Human NK Cell Deficiency and Their Effect on NK Cell Subsets

**DOI:** 10.3389/fimmu.2016.00545

**Published:** 2016-12-02

**Authors:** Emily M. Mace, Jordan S. Orange

**Affiliations:** ^1^Center for Human Immunobiology, Baylor College of Medicine and Texas Children’s Hospital, Houston, TX, USA

**Keywords:** NK cell deficiency, NK cell development, NK cells, primary immunodeficiency, immune homeostasis

## Abstract

Human NK cells play critical roles in human host defense, particularly the control of viral infection and malignancy, and patients with congenital immunodeficiency affecting NK cell function or number can suffer from severe illness. The importance of NK cell function is particularly underscored in patients with primary immunodeficiency in which NK cells are the primary or sole affected population (NK cell deficiency, NKD). While NKD may lead to the absence of NK cells, we are also gaining an increasing appreciation of the effect that NKD may have on the generation of specific NK cell subsets. In turn, this leads to improved insights into the requirements for human NK cell subset generation, as well as their importance in immune homeostasis. The presence of inherently abnormally developed or functionally impaired NK cells, in particular, appears to be problematic in the way of interfering with normal human host defense and may be more impactful than low numbers of NK cells alone. Here, we review the known genetic causes of NKD and the insight that is derived by these into the requirements for human subset generation and, by extension, for NK cell-mediated immunity.

## Introduction

While human NK cells and NK cell subsets have been well described, understanding the true physiologic role that they serve in humans has been difficult. The heterogeneity of NK cells as a result of both genetic and environmental diversity, as well as the divergence in phenotypic markers between mouse and man has made advances in the field incremental. An important tool in the understanding of human NK cells and NK cell subsets has been the discovery of human inborn genetic immunodeficiency diseases (primary immunodeficiencies) that affect the generation or homeostasis of NK cells or specific NK cell subsets as well as those that affect NK cell function. There are over 300 genetic deficiencies of human immunity, and nearly 50 are known to have at least some impact on NK cells. This latter category has been reviewed by a number of authors in recent years (1–4). Five of these primary immunodeficiencies, however, may have their primary impact upon NK cells and are known as NK cell deficiency (NKD) (these are summarized in Table 1). These have provided insight into the requirements for human NK cell maturation, the importance of the successful execution of NK cell development, and the overall value of certain NK cell functions. Here, we will describe recent advances in our understanding of the importance of human NK cell subsets and NK cell function as informed by NKD.

**Table 1:** **Human NK cell deficiencies and their effect on NK cell subsets, function, and proliferation**.

NKD type	Gene	Inheritance	NK cell number	NK cell lytic function	NK cell proliferation	CD56^dim^	CD56^bright^	Associated immune phenotype	Reference
cNKD	*GATA2*	AD or spontaneous	Normal or ↓	↓	ND	Normal or ↓	Absent/severely ↓	DC cytopenia, B cell lymphopenia, monocytopenia, CD4 lymphocytopenia, neutropenia (all variable across reported cases)	(33, 37–39)
cNKD	*IRF8*	AR	↓	↓	ND	↓	Normal/↑	Mild DC phenotype	(66)
cNKD	*MCM4*	AR	↓	↓	↓	↓	Normal/↑	None	(53, 54)
cNKD	*RTEL1*	AR	↓	↓	Normal	ND	ND	None in NKD patient reported	(60, 61)
fNKD	*FCGR3A*	AR	Normal	↓	ND	Normal	Normal	None	(71)

*AR, autosomal recessive; AD, autosomal dominant; ND, not done*.

## Human NK Cell Subsets

Human NK cells in peripheral blood are broadly classified as being CD56^+^CD3^−^ and generally comprise 1–17% of mononuclear cells within peripheral blood of healthy adults (5). They are found primarily in two broadly defined phenotypic subsets, namely the CD56^bright^ and CD56^dim^ subsets, with the CD56^dim^ subset being the predominant one in peripheral blood. While the basis for this stratification is the surface density of CD56 itself, each is a distinct functional subset with its own repertoire of markers that represent its capabilities. CD56^bright^ NK cells are broadly defined as being the potent producers of cytokines, whereas the CD56^dim^ subset is the classical mediator of contact-dependent cytotoxicity, both natural and antibody-dependent. However, exceptions to these rules exist, and there is evidence that both subsets can perform the tasks commonly attributed to the other (6, 7). In addition, there are several well-defined functional and phenotypic intermediaries between the two, as well as several subsets that are specifically expanded after infection or other immune stimulation. These include the CD56^neg^ subset, which is commonly found expanded in patients with HIV and HCV (8, 9). It should be noted that these cells are not truly CD56 negative, although the expression of CD56 on them is significantly lower than that of the CD56^dim^ subset. They retain expression of CD16, killer cell immunoglobulin receptors (KIR, CD158 family), and CD57 and are generally thought to be terminally differentiated or exhausted cells (10). An additional important subset expanded in disease is the adaptive NKG2C^+^ population expanded following certain viral infections, particularly CMV (11–13), but also in cases of reinfection of CMV seropositive individuals by hantavirus (14), HIV (15, 16), Epstein–Barr Virus (17), Hepatitis C Virus (18), and Chikungunya virus (19). Further studies have revealed distinct subsets associated with the generation of adaptive NK cells following CMV infection. These cells are high affinity IgE receptor (FcɛRIγ)-deficient and express low amounts of Syk and/or EWS/FLI1-activated transcript 2 (EAT2) (20, 21). Further adaptation of the NK cell repertoire occurs with epigenetic silencing of promyelocytic leukemia zinc finger (PLZF) (22). There is also remarkable diversity generated by both environmental and genetic factors within the human population as a whole with regards to NK cell receptor expression and functional potential (23, 24).

The relationship between CD56^bright^ and CD56^dim^ subsets is not entirely clear, although it is thought that they represent the two terminally mature populations of NK cells derived from less mature NK cell precursors. There are oft-cited lines of evidence suggesting that CD56^bright^ NK cells are the direct precursors of the CD56^dim^ subset, namely the longer telomeres in CD56^bright^ NK cells (25), their earlier regeneration after HSCT (26), and the generation of CD56^dim^ NK cells from CD56^bright^ cells in certain experimental systems (27–29). The expression of CD56 on NK cells is unique to human and non-human primate systems, which has made the study of this specific aspect of NK cell development difficult to model prior to the advent of humanized mouse models. The use of such humanized mice, in which human NK cells develop in a rodent system, showed that CD56^bright^ NK cells give rise to CD56^dim^ cells (29); however, a recent study using genetic bar coding of NK cell lineages in rhesus macaques was indicative of the two subsets having distinct ontologies (30). Ultimately, answering this important question for NK cell biology will require a dissection of the mechanism that drives NK cell development using a satisfying human model. The study of primary human NKDs that affect NK cell subset generation are an informative tool in this endeavor, specifically for identifying novel requirements for the generation and homeostasis of human NK cell subsets (discussed further below). While complex, these disorders also provide insight into the functions of particular NK cell subsets in humans, as well as the overall value of NK cells in human host defense.

## Classical NKD

Classical (c)NKD refers to NKD as a result of absent or profoundly decreased CD56^+^ CD3^−^ NK cell number, specifically defined as ≤1% of peripheral lymphocytes (3, 31). As a result, these are disorders in which overall NK cell development and/or homeostasis is impaired. Recent studies enabled by next generation sequencing techniques have identified genetic causes for previously described but unidentified cNKD. In combination with these genetic advances, we have also seen an increase in our understanding of NK cell subsets and the cell biological tools used to evaluate them. As a result, we can now appreciate that cNKD also includes cases in which human peripheral blood NK cell subsets are selectively underrepresented or absent. Thus, while cNKD has been historically used to define individuals with very low percentages of NK cells among peripheral blood lymphocytes, we would propose that this definition be expanded to include any example of impaired development or survival leading to the gross underrepresentation of a major NK cell subset. We would also argue that abnormally developed NK cells or the absence of particular NK cell subsets in the presence of others is far more relevant to host defense than simply having low overall numbers of NK cells.

To date, there are four genetically defined reported causes of cNKD, and of these at least three (*MCM4, GATA2, IRF8*) contain an aspect of subset aberration, in addition to NK cell numbers ranging from the low end of normal to undetectable. The fourth (*RTEL1*) presents information too limited to make a definitive statement other than there being underrepresented NK cells seemingly in isolation from other immune defects. In some cases, namely GATA2 deficiency, a single gene defect may account for a widely variable clinical phenotype with regards to NK cell number. This likely is related to the effect of specific mutations but may also be reflective of innate plasticity of NK cell numbers and subsets over time, in even healthy individuals, and the infectious history of the patient (5, 23, 24).

### *GATA2* 

GATA2 deficiency is the genetic lesion behind the most frequently cited case of classic NKD in the literature. A 13-year-old girl, originally presenting with varicella pneumonia, was found to have decreased NK cell number and function in the presence of T cell and B cell function (32). In addition to VZV, CMV and HSV infections followed, and she ultimately developed aplastic anemia and died during the course of hematopoietic stem cell transplantation (32, 33). Posthumous sequencing from a cryopreserved T cell line identified a pathogenic frameshift GATA mutation (c.1025_1026insGCCG; p.A342GfsX41), confirming GATA2 haploinsufficiency as the cause of her disease (33). GATA2 haploinsufficiency was also described as a cause of DC and monocyte deficiency (MonoMAC, DCML) by independent groups in 2009, and NK cell cytopenia was noted as a feature of disease (34–36). More detailed phenotyping has revealed that there is variation in the NK cell number in these patients, with many of them having less than 1% NK cells within their lymphocyte population. However, there is a substantive range in NK cell number, with some patients having NK cells within the normal range of healthy donors. Despite the presence of NK cells in some patients, a consistent and notable feature of GATA2 deficiency is the absolute loss of the CD56^bright^ NK cell subset (33). Originally described in a cohort of eight patients, this has since been reported by other groups (37, 38) and is a consistent feature of the >30 patients that we have studied with GATA2 deficiency and accompanying infectious history or hematologic disease.

While it is difficult to estimate the prevalence of GATA2 mutation within the general population, the report of a cohort of 57 patients, of which 82% have clinically detected NK cell lymphopenia and 70% have severe and early onset viral infection suggestive of NK cell dysfunction, is the largest group of NKD patients that we are aware of (39). This suggests it is perhaps the most common NKD, as this cohort alone significantly outnumbers those reported with *RTEL1, MCM4, IRF8*, and *FCGR3A* mutations. Furthermore, *GATA2* is also the only gene associated with NKD that is autosomal dominant (*via* haploinsufficiency) and the only for which spontaneous cases have been reported (35, 36), thus supporting the statement of commonality from a genetic standpoint as well.

GATA2 deficiency is complex as it can be a multi-syndromic disease affecting multiple organs and presenting in multiple different ways. Patients can be susceptible to atypical mycobacterial infections, fungal infections, and severe and recalcitrant viral infections. Interestingly, there is a seemingly progressive nature of the disease, with many patients presenting in young adulthood and some even later in life. The range of clinical presentations and natural history has been well described elsewhere (39). Interestingly, one of the earliest clinical features to appear is a susceptibility to HPV disease, which could point to a role for NK cell-mediated defenses. In addition to immune deficiency, GATA2 deficiency is a cause of familial bone marrow failure (40), and a recent study of over 400 children and adolescents revealed GATA2 mutation to be the most common germline mutation leading to myelodysplastic syndrome in children and young adults (41). While this group did not specifically examine NKD in their cohort, they report half of their patients to have immunodeficiency, suggesting that the high rates of NK cell cytopenias reported by Spinner et al. (39) are likely present in this group.

However, there are some notable aspects of GATA2 deficiency with regards to NK cell biology. While other NK cell deficiencies have been reported as affecting the frequency of subset distribution (particularly MCM4, described below), the loss of the CD56^bright^ NK cell subset is as near to absolute as has been described. The mechanism by which GATA2 regulates the development or maintenance of the CD56^bright^ NK cell pool is not understood. GATA2 is a zinc finger transcription factor that is required for embryonic hematopoiesis, and maintenance of the stem cell pool in adults (42, 43) and GATA2 haploinsufficiency can also lead to loss of dendritic cell subsets and B cell cytopenias. Therefore, given its important role in stem cell maintenance and hematopoiesis, GATA2 deficiency may affect multiple immune cell lineages, which are again highly variable from patient to patient. *In vitro* differentiation of NK cells from patient hematopoietic stem cells leads to aberrant NK cell development, suggesting that NKD is cell intrinsic in these patients (33). However, given the interdependence on particularly NK and DC cross talk, it is likely that loss of other subsets affects NK cell numbers and functions in these patients, although this has not been explicitly studied. Immune manifestations include susceptibility to mycobacterial disease, frequently *M. avium* and *M. kansasii*, susceptibility to fungal infection, and severe herpesviral infections (39). An increased rate of malignancy may be in part due to viral infection as this includes increased rates of cervical cancer in young women potentially attributable to human papillomavirus infection. Less common clinical symptoms include miscarriage, solid organ tumors, and lymphedema (Emberger’s syndrome) (39).

Due to the requirement for GATA2 in renewal of the adult stem cells, it is thought that depletion of the stem cell pool leads to subsequent lymphopenia, and *in vitro* differentiation experiments demonstrate an NK cell intrinsic role for GATA2 through the phenocopying of the CD56^bright^ NK cell subset loss in NK cells derived from patient CD34^+^ HSC (33). Whether GATA2 is required for the generation or homeostasis of CD56^bright^ NK cells, or whether the defect arises earlier in lineage commitment, remains to be determined. GATA2 is highly expressed not only in CD34^+^ HSC, but also across a range of lineages including monocytes, monocyte-derived dendritic cells, B cells, and mature NK cells, as well as the common myeloid precursor (44–46). Conditional deletion of *Gata2* in mice has recently demonstrated its requirement for DC differentiation from lineage negative precursors (47). Interestingly, this is at least partially through repression of genes that control T cell and ILC lineages, including *Tcf7, Eomes*, and *Gata3*. Direct binding of the Gata3 promoter by Gata2 in common myeloid progenitor cells suggests that these two transcription factors may play a role in tuning fate decisions similar to the well described GATA switch that occurs between GATA1 and GATA2 and is independent of transcription factor expression (47, 48).

This particular deficiency also allows for one to hypothesize regarding potential contribution of NK cells to human host defense. Given that some GATA2 deficiency patients lack only the CD56^bright^ subset, it could speak to an important role for human NK cell-derived cytokine production specifically in the defense against HPV and/or herpesviruses. As there is strong animal model support for this role (49–51), the connection is at least plausible. It is not possible, however, to draw a direct connection as there is the variable impact upon other elements of innate immunity in these patients as discussed above. Furthermore, there are also those GATA2 deficiency patients that essentially lack NK cells altogether as well as those who have CD56^dim^ NK cells. As deficient natural cytotoxicity is a conserved clinical hallmark of GATA2 deficiency (32, 33), this leaves open the contribution of contact-dependent cytotoxicity as the critical NK cell-mediated component of viral control. Thus, while GATA2 deficiency provides both insights and compelling leads, further work needs be done to formally attach a specific role to NK cell defenses through this disorder.

### *MCM4* 

Mutations in minichromosomal maintenance complex member 4 (MCM4) were described in 2012 as a cause of NKD accompanied by adrenal insufficiency, developmental delay, and short stature in a population of endogamous Irish travelers (52–55). Notably, patients in this cohort had increased susceptibility to viral infection, including cytomegalovirus, and malignancy (52–54). An initial description selective NKD (52) was followed by a genetic diagnosis of splice-site mutations in MCM4 and more detailed analysis of the NK cell phenotype (53, 54). MCM4 patients were the first to have demonstrated heritability to NKD and were the first listed in the Online Mendelian Inheritance in Man database as such (OMIM# 609981). Patients with MCM4 mutation have decreased frequency of CD56^dim^ NK cells and, as a result, a relative overrepresentation of the CD56^bright^ subset with an overall decrease in absolute numbers of NK cells (53). Careful analysis of both NK cells and fibroblasts from six patients in the Irish traveler cohort yielded novel insight into both NK cell biology and the role of the MCM complex in NK cell homeostasis and human disease. Specifically, CD56^bright^ NK cells from patients had decreased rates of proliferation and impaired terminal maturation. This likely accounts for the severe viral susceptibility in these patients as they are lacking the more mature subset of NK cells and have at least some instability within the CD56^bright^ population (53).

Why does MCM4 mutation exert such a specific, profound effect on NK cell maturation and function? MCM4, along with MCM2–7, MCM10, and GINS1 form a highly conserved complex that plays a critical role in the initiation and elongation of eukaryotic DNA replication (56, 57). Embryos from the Mcm4 knockout mouse do not survive implantation, and mice carrying the hypomorphic *Chaos3* mutation affecting Mcm4 have genomic instability reminiscent of that found in MCM4 patients (58). Careful analysis of the patients’ fibroblasts showed normal MCM2–7 complex formation and DNA-binding but impaired DNA replication and, subsequently, cell cycle arrest (53). Genomic instability frequently accompanies impaired replication, and this is also the case in MCM4 patient fibroblasts, which had increased rates of chromosomal breakage (53). Interestingly, accompanying the decrease in NK cell number and specific decrease in frequency of CD56^dim^ NK cells, patient NK cells had impaired proliferation in response to cytokine stimulation and increased rates of apoptosis (53).

The apparent role of MCM4 in the generation of CD56^dim^ NK cells is not fully understood; however, it may speak to a previously hypothesized requirement for homeostatic proliferation, specifically in the CD56^bright^ subset, leading to generation of the CD56^dim^ subset. This model was originally proposed based on differential rates of apoptosis and proliferation detected in CD56^bright^ and CD56^dim^ NK cells in peripheral blood (59). The rapid proliferation of peripheral blood CD56^bright^ cells with little apoptosis suggests that this subset either becomes recruited to tissue or generates the CD56^dim^ subset, as substantiated by mouse xenograft studies (29). Should the latter be the case, the loss of CD56^dim^ NK cells in MCM4 patients could be explained by the inability of the CD56^bright^ NK cells to successfully undergo this proliferative burst and effectively generate CD56^dim^ NK cells. Clearly, MCM4 deficiency raises many important new questions in NK cell biology, and answers will undoubtedly provide leads into both how NK cells develop as well as how developing NK cells figure into human host defense.

### *RTEL1* 

The third case of classic NKD, similarly to GATA2, is a case of a young girl who died of varicella infection at the age of 2 years. Originally reported in 2005 (60), her genetic lesion was recently solved by whole exome sequencing and found to be due to homozygous mutation in *RTEL1* (61). Consistent with RTEL1 being a cause of specific NKD, the girl had seemingly normal immunoglobulin levels and specific antibody titers, normal T and B lymphocyte counts and subsets, and absence of bone marrow failure. Notably, however, she had severely decreased NK cell number and function that was not rescued by IL-2. As common γ chain and JAK3 mutations lead to SCID, her IL-15 levels and signaling pathways were tested, yet were found to be intact (61). *RTEL1* deficiency is a reported cause of Hoyeraal–Hreidarsson Syndrome, an X-linked telomere deficiency prevalent among the Ashkenazi Jewish population that causes dyskeratosis congenita, bone marrow failure syndrome, and immunodeficiency (62). The mutation reported in this (2-year-old female) patient is a founder mutation causing Hoyeraal–Hreidarsson syndrome and lymphopenia, including progressive NK cell immunodeficiency (63, 64). Interestingly, the patient had no clinical features of Hoyeraal–Hreidarsson syndrome, and her growth and neurological development were normal for her age (personal communication, A. Etzioni, 2016). She did have an inverted CD4/CD8 T cell ratio but was virally infected (61). The patient also did not have recognizable platelet abnormalities or anemia (personal communication, A. Etzioni, 2016). Since the discovery of this patient, there have not been other reports of biallelic *RTEL1* mutations causing selective NKD. However, given the molecular mechanism of MCM4 deficiency, the link between RTEL1, which also plays a role in DNA repair, and NK cell development is interesting. As the patient was deceased at the time of genetic study, unfortunately in-depth studies of NK cell subsets or developmental processes were not tenable. The effect of RTEL1 deficiency on NK cell development underscores the specific sensitivity of human NK cells to DNA damage and the requirement for the DNA damage response in normal human NK cell development and homeostasis.

### *IRF8* 

The most recent description of cNKD identifies the cause of NKD in a family first described in 1982 with severe EBV susceptibility and absent NK cell function in affected individuals (65). The longitudinal study of a surviving affected sibling shows a distinctive, stable NK cell phenotype, with decreased NK cell function and increased CD56^bright^ NK cells relative to the CD56^dim^ subset (66). This phenotype is conserved in other patients with biallelic IRF8 mutation and is NK cell intrinsic. Gene expression analysis of NK cells from the proband shows deregulation of genes involved in NK cell maturation and effector function, suggesting that IRF8 is playing a role in the regulation of genes that include *NFIL3, PRDM1, TBX21, GRZB, STAT5a, STAT5b*, and *PRF*. Strikingly, a similar block in terminal maturation is also observed in mice with homozygous, but not heterozygous *Irf8* mutations. IRF8 deficiency as a result of both homozygous and heterozygous mutation can lead to DC deficiency (67, 68), although DC subsets in the proband reported here were only minimally affected (66). While mutation and gene dosage effects may be at play, the NK cell phenotype was conserved between all patients with biallelic IRF8 mutations studied but was not identified in any individuals with heterozygous IRF8 mutation. These findings demonstrate a requirement for IRF8 in terminal NK cell maturation and human antiviral defense and identify biallelic mutations in IRF8 as a newly described cause of cNKD.

## Functional NKD

In contrast to cNKD, functional NKD (fNKD) represents a scenario in which NK cells are present in normal numbers but have some impaired functional capacity. In fNKD, a patient’s NK cells appear to have gone through appropriate developmental progression and are not unduly susceptible to cell death (i.e., impaired survival). This distinction makes this category particularly difficult to diagnose clinically as the most common screening tests of quantitative flow cytometry will most likely be normal. Thus, a suspicion given a patient’s particular clinical susceptibility is necessary to prompt a more substantive assessment of NK cell function. While presently fewer in number, it is possible that the fNKD category may ultimately outweigh the cNKD category in number of mechanistically defined diagnoses. Presently, there is only one known fNKD that is caused by biallelic specific mutations in *FCGR3A*.

### *FCGR3A* 

The only cause of fNKD reported to date is a rare mutation in *FCGR3A* (OMIM #615705), the gene encoding the low affinity IgG Fc receptor found on NK cells and macrophages (FcγRIIIA, CD16). Originally reported as the first monogenic cause of isolated NKD (69, 70), the c.230T-A transversion leads to L66H substitution in the first extracellular Ig domain of CD16 and is disease-causing when homozygous (69–71). Notably, while protein is expressed, the L66H substitution leads to loss of recognition of CD16 by mAb B73.1 while retaining recognition by the more commonly used 3G8 mAb (71). Therefore, dual detection with these antibodies is a rapid screen for this rare mutation as in certain individuals the B73.1 epitope will not be detected, although genotyping is required to confirm this molecular diagnosis (as individuals have been identified lacking the epitope for other reasons). Aside from the apparent loss of CD16^+^ NK cells if using mAb B73.1, NK cells from these patients appear to have appropriate development and subset generation (71).

This cause of fNKD was first identified in two patients with recurrent frequent upper respiratory infections and recurrent HSV (as well as herpes whitlow in one patient and recurrent varicella zoster in the second) (69, 70). Our group subsequently identified homozygous L66H substitution in a 14-year-old male with recurrent EBV-driven Castleman’s disease and recalcitrant cutaneous warts (71). While CD16 function is required to mediate ADCC, strikingly the patients with CD16 L66H mutation have normal ADCC-mediated cellular cytotoxicity, yet impaired natural cytotoxicity. This seemingly paradoxical effect is explained by the location of the mutation in the membrane distal Ig-like domain, which does not affect binding to IgG Fc mediated by the membrane proximal domain. Instead, molecular studies of the effect of L66H mutation revealed a role for the distal Ig-like domain of CD16 in binding to and stabilizing the NK cell coactivating receptor CD2, thus delineating a crucial role for CD16 in the co-stimulation of natural cytotoxicity in addition to its well-defined role in ADCC (71). Recently, the biology of this interaction has been further advanced through the study of individuals who do not possess the NKG2C locus in which a compensatory co-stimulatory relationship between CD2 and CD16 were demonstrated to be of value (72). Thus while, only one example of fNKD, this disorder has provided valuable insights into how NK cells react to challenges and focus upon patients with abnormal NK cell function, but grossly normal appearing NK cells should lead to important new leads both biologically and clinically.

## Putting it all in Context

While originally classified as classical or functional, the lines between the types of NK cell deficiencies has been blurred by increased resolution of NK cell subsets as well as the normative ranges and diversity of human NK cells (5, 23, 24). This is exemplified by the example of GATA2 mutation, which was initially reported as leading to absence of circulating NK cells but which, upon careful analysis was shown to lead to decreased NK cell number, specifically loss of the CD56^bright^ NK cell subset, accompanied by decreased function (33). Thus, it is possible to have a scenario in which NK cells may be present by clinical enumeration in a patient with classical NKD. The use of multiparametric flow cytometry to evaluate even the most rudimentary of NK cell subsets provides invaluable insight into the potential source of NKD. This is particularly true of GATA2 deficiency, which has a distinctive and seemingly immutable phenotype with regards to the absence of CD56^bright^ NK cells (33, 37, 38).

A fascinating biological question has emerged from these discoveries of NKD, namely that of the interdependence of subsets upon each other for function, as well as the relative importance of NK cell number versus functional potential. Particularly in the case of GATA2 and MCM4, the skewing of CD56^bright^ and CD56^dim^ subset generation has a seemingly profound effect on the function of the other subset (33, 53). Namely, in cases where CD56^dim^ NK cells are present in GATA2 patients, these cells remain unable to mediate the contact-dependent target cell lysis that is a functional hallmark of the CD56^dim^ subset. In the case of MCM4 deficiency, although it seems that the CD56^bright^ subset is impacted more so than the CD56^dim^ subset, profoundly decreased NK cell function by the CD56^dim^ subset is a likely a contributing cause of the patients’ disease (54).

These observations add to an interesting but complicated literature on the ontology of human NK cells and ultimately speak to the need for a better understanding of the mechanism of, and requirement for human NK cell development. Finally, the question of the relationship between NK cell number, subset distribution, function, and disease state remains incompletely understood. Whether it is more advantageous to have fewer NK cells that are appropriately developed, as opposed to a greater number of inappropriately mature cells, remains to be seen. The evidence points to the former, begging the analogy that it is more important to have a small, functional team than a large one with badly behaved players. By extension, a small team may be a result of a disruptive individual, so determining the relative contribution of the deregulation of function and/or phenotype will be increasingly insightful into the pathogenesis of NKD. Emerging evidence in transplantation for primary immunodeficiency emphasizes this point, as having potentially very low numbers of NK cells that have the potential to work effectively may suffice at the right time and under the right circumstance. Along these lines, it is even possible that having a few defective NK cells could be more damaging than having none at all. Answering these questions will require further studies that carefully and thoughtfully dissect human NK cell development in health and disease. Furthermore, there are likely to be context-, time-, and exposure-specific components of NKD as there are likely to be critical windows in which an absence of NK cell subsets or functions may be particularly relevant for human host defense.

It should also be noted that all these studies in NKD will benefit from a greater understanding of NK cells within unique microenvironments. By necessity, most of this work has used peripheral blood as the source of NK cells, yet the tissue-specific NK cell phenotypes in liver, gravid uterus, and secondary lymphoid tissue speak to their shaping by these sites. Understanding how these seldom-visualized cells are affected by disease will be an important component of a greater understanding of the non-redundant function of human NK cells in health and disease. The present state of knowledge, however, suggests that there are patients with deficiencies of immunity reflected primarily within the NK cell compartment who are in need of mechanistic and therapeutic answers. Further study into individuals with suggestive phenotypes will likely produce new causative genes for NKD and insights into NK cell biology that can ultimately lead to new and improved therapeutic directions.

## Author Contributions

Both the authors contributed equally to the writing and editing of this manuscript.

## Conflict of Interest Statement

The authors declare that the research was conducted in the absence of any commercial or financial relationships that could be construed as a potential conflict of interest.
